# The Role of Interferon-γ Inducible Protein-10 in a Mouse Model of Acute Liver Injury Post Induced Pluripotent Stem Cells Transplantation

**DOI:** 10.1371/journal.pone.0050577

**Published:** 2012-12-05

**Authors:** Che-Chang Chan, Ling-Yi Cheng, Jean Lu, Yi-Hsiang Huang, Shih-Hwa Chiou, Ping-Hsing Tsai, Teh-Ia Huo, Han-Chieh Lin, Fa-Yauh Lee

**Affiliations:** 1 Institute of Clinical Medicine, School of Medicine, National Yang-Ming University, Taipei, Taiwan, Republic of China; 2 Institute of Pharmacology, School of Medicine, National Yang-Ming University, Taipei, Taiwan, Republic of China; 3 Genomics Research Center, Academia Sinica, Taipei, Taiwan, Republic of China; 4 Division of Gastroenterology, Department of Internal Medicine, Taipei Veterans General Hospital, Taipei, Taiwan, Republic of China; 5 Stem Cell Lab, Department of Medical Research and Education, Taipei Veterans General Hospital, Taipei, Taiwan, Republic of China; Beijing Institute of Infectious Diseases, China

## Abstract

**Background:**

Liver injuries are important medical problems that require effective therapy. Stem cell or hepatocyte transplantation has the potential to restore function of the damaged liver and ameliorate injury. However, the regulatory factors crucial for the repair and regeneration after cell transplantation have not been fully characterized. Our study investigated the effects and the expression of the regulatory factors in mouse models of acute liver injury either transplanted with the induced pluripotent stem cells (iPS) or the hepatocytes that differentiated from iPS cells (iHL).

**Methods/Principal Findings:**

Mice received CCl_4_ injection and were randomized to receive vehicle, iPS, or iHL transfusions vial tail veins and were observed for 24, 48 or 72 hours. The group of mice with iPS transplantation performed better than the group of mice receiving iHL in reducing the serum alanine aminotransferase, aspartate aminotransferase, and liver necrosis areas at 24 hours after CCl_4_ injury. Moreover, iPS significantly increased the numbers of proliferating hepatocytes at 48 hours. Cytokine array identified that chemokine IP-10 could be the potential regulatory factor that ameliorates liver injury. Further studies revealed that iPS secreted IP-10 *in vitro* and transfusion of iPS increased IP-10 protein and mRNA expressions in the injured livers *in vivo*. The primary hepatocytes and non-parenchyma cells were isolated from normal and injured livers. Hepatocytes from injured livers that received iPS treatment expressed more IP-10 mRNA than their non-hepatocyte counter-parts. In addition, animal studies revealed that administration of recombinant IP-10 (rIP-10) effectively reduced liver injuries while IP-10-neutralizing antibody attenuated the protective effects of iPS and decreased hepatocyte proliferation. Both iPS and rIP-10 significantly reduced the 72-hour mortality rate in mice that received multiple CCl_4_-injuries.

**Conclusions/Significance:**

These findings suggested that IP-10 may have an important regulatory role in facilitating the repair and regeneration of injured liver after iPS transplantation.

## Introduction

Liver diseases and injuries are important medical problem worldwide. Liver transplantation is currently the most efficient therapy for liver failure and end-stage liver disease. However, it is limited by the scarcity of donor, expensive medical cost, surgical risk and requiring life-long immunosuppressant agents. The development and application of hepatocytes transplantation has been attempted to treat different forms of liver diseases [1], [2], [3]. It has minimal invasive procedures and fewer surgical complications compared to the orthotopic liver transplantation. Stem cell transplantation has also gained considerable attention recently. Stem cells have the potential to supportive tissue regeneration and to generate large amounts of donor cells ready for transplantation [4], [5], [6], [7].

The induced pluripotent stem cells (iPS) are generated from differentiated cells by genetic reprogramming technique [8]. They possess the abilities to self-renew and differentiate into different cell types after proper induction [8], [9], [10]. The major advantage of iPS is that they can be generated from somatic cells. The use of autologous iPS avoids immune rejection after transplantation and the ethical concerns raised by using embryonic stem cells. In recent years, the potential roles of iPS or the hepatocytes that differentiated from iPS in the management of liver injury have recently gained increasing attention [7], [11], [12].

Although previous studies using stem cells in treating liver injuries have shown beneficial effects [13], [14], [15], the underlying mechanisms for their therapeutic effects have not been completely revealed. One possible explanation is that the transplanted stem cells generate cells function well as normal hepatocytes do. However, the percentage of engraftment and graft survival after cell transplantation remains disappointing [2], [16]. Another explanation is the indirect paracrine effects initiated in the damaged liver after stem cell transplant [14], [17]. Some soluble factors such as cytokines or chemokines may have been secreted in order to facilitate the process of damage repair and liver regeneration. One of the CXCR3-related chemokines, the interferon-γ-inducible protein 10 (IP-10), has been regarded as a marker of inflammatory damage. Besides, IP-10 expression was shown to correlate with the degree of liver inflammation, necrosis and fibrosis [18], [19], [20]. Recently it was found that IP-10 could modulate either positively or negatively the repair and the regeneration process in different forms of liver injuries [21], [22], [23], [24]. Thus, we proposed that IP-10 may have an important regulatory role in the cell-base therapy for acute liver injury. At present, it remains unclear whether or not IP-10 is involved in and regulates the recovery process of the injured liver after stem cell transplantation.

In this study, we induced iPS to differentiate into hepatocytes *in vitro*. These cells were referred as iPS-derived hepatocyte-like cells (iHL), which functionally resemble primary hepatocytes. Then we investigated the effects of iPS and iHL on acute toxin-induced liver injury and explored the possible underlying paracrine-mediated mechanism. Here, we showed that transplanted iPS increased the expression of IP-10 in injured liver to facilitate damage repair and promote liver regeneration.

## Results

### iPS Alleviated Liver Injury and Promoted Regeneration

**Figure 1:**
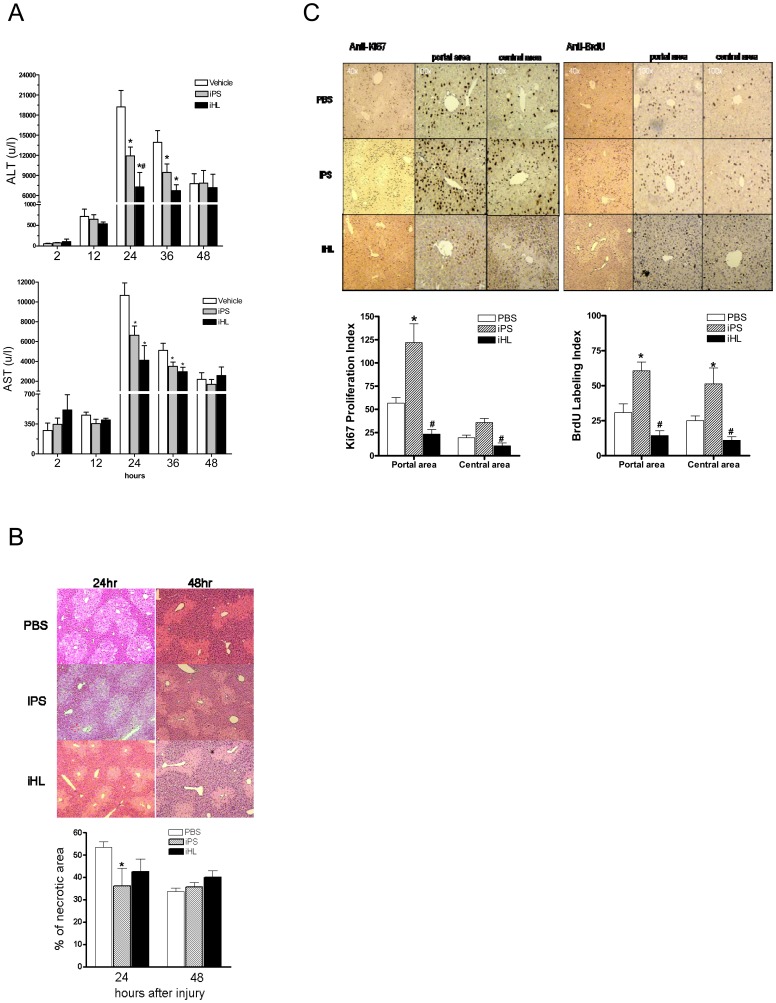
iPS and hepatocytes transplantation reduced hepatic injury. (**A**) Mean AST and ALT levels in mice receiving PBS (open bars), iPS (gray bars), and iHL (solid bars) following CCl_4_ treatment (n = 6, *P<0.05 vs. PBS, ^#^P<0.05 vs. iPS). (**B**) Representative liver sections from CCl_4_-injured mice that received vehicle, iPS or iHL infusion. Necrotic area were quantified and the percentage were shown (n = 5, *p<0.05 vs. vehicle). (**C**) At 48 h post CCl_4_ treatment, hepatocyte proliferation of vehicle (PBS), iHL, iPS was measured by Ki67 immunostaining and BrdU incorporation assay (n = 6, *p<0.05 vs. PBS, ^#^ *p*<0.05 vs. iPS).

To establish a liver-injury animal model, we injected mice with CCl_4_ and evaluated the degree of hepatocyte injury by measuring ALT and AST. As shown in Figure 1A, the CCl_4_-injured mice showed peak levels of serum ALT and AST at 24 hours. Infusion of iPS or iHL cells significantly decreased ALT and AST levels at 24 and 36 h following CCl_4_ treatment (n = 6, P<0.05) (Figure 1A). The liver histology revealed that CCl_4_ induced a submassive centrilobular necrosis, which presented with light colors (Figure 1B). iPS infusion reduced the necrotic percentage by 40% compared to the control group. In contrast, iHL infusion did not significantly lesson the degree of necrosis (P>0.05). We next investigated the hepatocyte proliferation, which is a critical sign of liver regeneration (Fig. 1C). BrdU incorporation was used to quantify hepatocytes in S phase. Positive immunostaining of Ki67 represents the hepatocytes in cell cycle progression. Only few proliferating hepatocytes were detected by anti-Ki67 and anti-BrdU antibodies in all three groups at 24 h post-injury (data not shown). Differences in the proliferative response became obvious at 48 h. More than two folds of the proliferating hepatocytes were present in the iPS group compared to the controls. In contrast, the number of proliferating hepatocytes in iHL group was significantly lower than iPS group, indicating that only iPS have potential to promote liver regeneration.

### Localization of iPS in the Injured Liver

**Figure 2:**
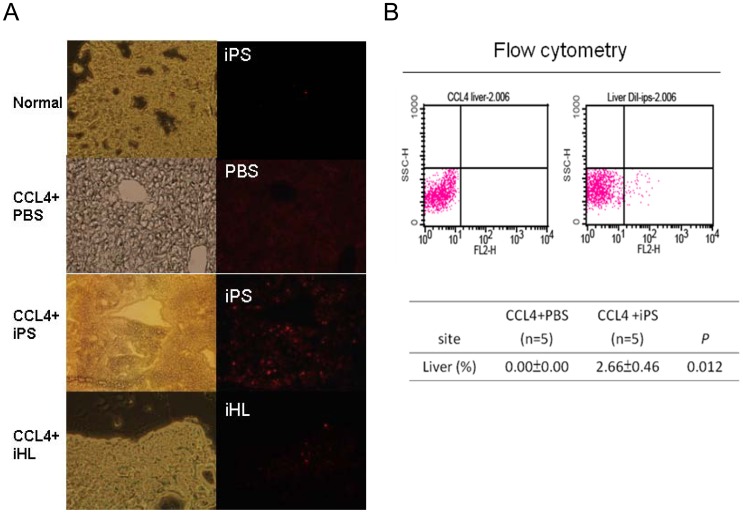
Localization of iPS in injured liver. The iPS and iHL were labeled with a red fluorescent dye (DiI) before use. (**A**) At 24 h post-injury, frozen sections of livers from different groups were observed. The background of Red fluorescent was present at the PBS control. The strong red fluoresence signals indicate the iPS or iHL localized in the liver. (**B**) The representative flow-cytometry diagrams showed that iPS localized in liver. Flow cytometry assay were used to calculate the percentage of iPS engrafted in the liver.

From above results, iPS outperformed the iHL in promotion of hepatocyte regeneration. Therefore, we further examined the engraftment of the transplanted iPS. To examine the localization of iPS in the liver, we labeled iPS with a red fluorescence dye, DiI, before infusion. Under fluorescent microscopic observation, the percentages of positive cells were highest in iPS group when compared to the control and iHL groups (Fig. 2A). Besides, significant numbers of fluorescent cells were detected in liver, spleen, lung, and bone marrow by flow-cytometry analysis (Fig. 2B & Table S2). The majority of the DiI-labeled iPS was localized in the liver and spleen and the mean percentages were 2.66% and 4.74%, respectively. This implied that the iPS may function in liver through a direct or an indirect route. Additionally, no iPS-induced teratoma was detected in the study mice during a 6-month observation period (Fig. S3).

### IPS Increased Hepatic IP-10 Expression in Injured Liver

**Figure 3:**
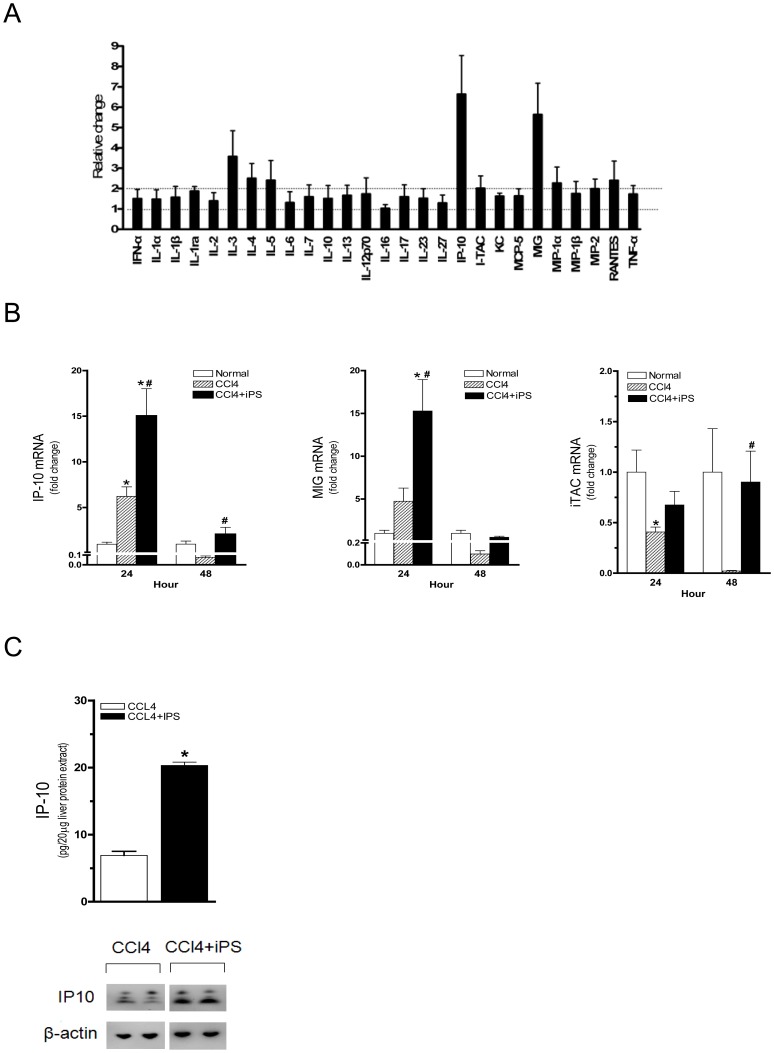
Changes of hepatic chemokines after iPS infusion in injured mice. (**A**) iPS-induced changes of cytokines in the liver were evaluated by cytokine array. (**B**) The hepatic expression of IP-10 and MIG were increased after CCl_4_ injury. iPS infusion further increase their expressions, but not for iTAC. At 48 h post-injury, the hepatic expression of IP-10 and MIG decreased but IP-10 remained at levels significantly higher than those of the CCl_4_ group (n = 6, *p<0.05 vs. normal control, ^#^ *p*<0.05 vs. CCl_4_ group). (**C**) Hepatic IP-10 at 24 h post-injury was measured in homogenized liver extract by ELISA and western blot. iPS infusion significantly increased hepatic IP-10 in CCl_4_-injured liver. (n = 4, *p<0.05 vs. normal control, ^#^ *p*<0.05 vs. CCl_4_ group).

The iPS-induced cytokines changes in the liver were evaluated by cytokine array (Fig. 3A). Among all the cytokines tested, IP-10 and MIG were upregulated by 7- and 6-folds in liver tissues respectively. Further study showed that the mRNA expression of IP-10 and MIG significantly increased at 24 h post-injury (Fig. 3B). In contrast, the expression of iTAC, which belong to the same cytokine family as IP-10 and MIG, decreased (Fig. 3B). At 48 h post-injury, the levels of IP-10 and MIG decreased, but the expression of IP-10 in the iPS group remained significantly higher than that in the CCl_4_ group without iPS treatment (*p*<0.05). At protein levels, results from ELISA and Western blot analysis demonstrated that there was a significant increase of hepatic IP-10 by iPS at 24 h post-injury (Fig. 3C).

### IPS and Hepatocytes as the Cellular Sources of IP-10

**Figure 4:**
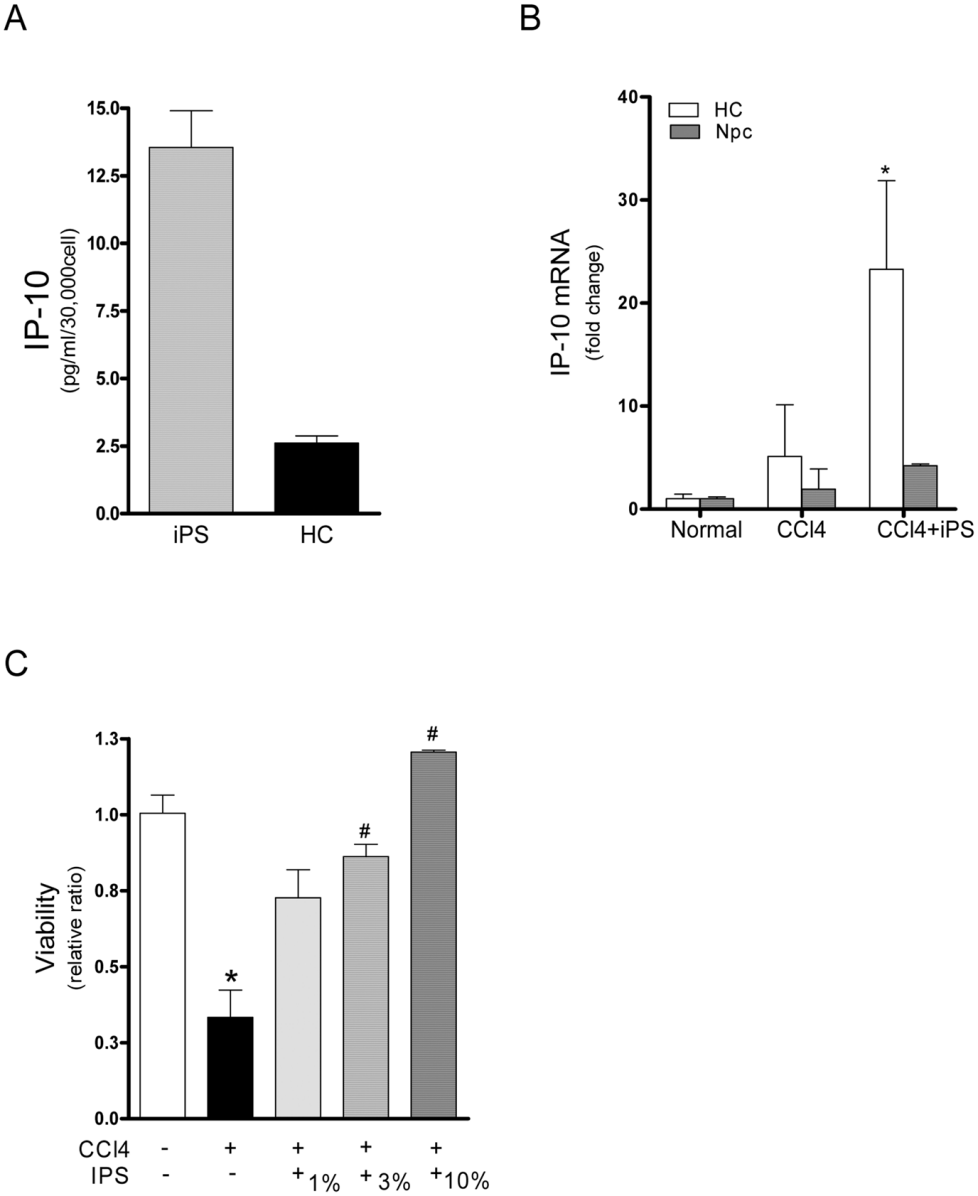
The cellular source and the beneficial effects of IP-10. (**A**) *In vitro* cultured iPS secreted IP-10 into culture medium. (**B**) Mice primary hepatocytes (HC) and none-parenchymal cells (Npc) were isolated from normal and injured mice livers at 24 h post-injury. After iPS infusion, increased expression of IP-10 mRNA were observed mainly in HC from injured liver after iPS treatment (n = 3). (**C**) Mice none-transformed hepatocytes (AML12) were co-cultured with iPS. iPS increased the viability of the CCl_4_-injured hepatocytes (n = 3 independent experiment).

Next we tested if iPS can secret IP-10 directly and be the source of IP-10 *in vivo*. The iPS was found to secrete IP-10 into culture medium at concentration about 14 pg/ml per 30,000 cells; while compatible number of hepatocytes (AML12) secreted IP-10 at concentration of 2.9 pg/ml only (Fig. 4A). In addition, the primary hepatocytes and non-parenchymal cell (Npc) were isolated from normal and the injured liver for their IP-10 expressions. After iPS infusion, an increased IP-10 mRNA expression was observed in primary hepatocytes from the injured liver (Fig. 4B). In *in vitro* co-culture study, increasing the numbers of iPS increased the viability of hepatocytes (AML12) (Fig. 4C). We also investigated whether the expression of two common IP-10 inducers, the IFN and TNF-α were positively correlated with IP-10 expression in the CCl_4_-injured mice. The results demonstrated that these common inducers were not responsible for the IP-10 induction (Fig. S4).

### IP-10 is an Important Factor that Modulate the Beneficial Effect of iPS

**Figure 5:**
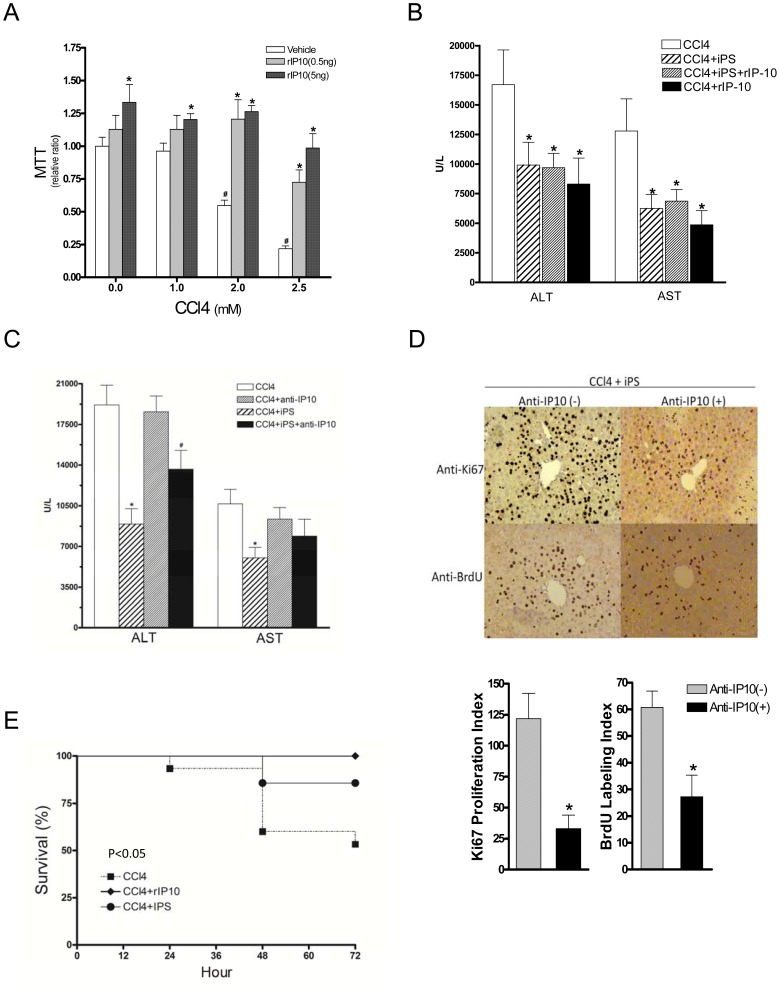
IP-10 is an important factor that mediated the beneficial effects of iPS. (**A**) Recombinant IP-10 (rIP-10) increased the viability of injured hepatocytes 24 h after CCl_4_ injury at concentration of 1.0 to 2.5 mM. (**B**) In injured mice, rIP-10 reduced the degree of liver damage and the effects of rIP-10 were compatible to iPS alone. Combined treatment of rIP-10 and iPS had no additional damage-reducing effects. (**C**) Anti-IP-10 was used to neutralize the effect of IP-10. Application of anti-IP-10 antibody itself did not exert significant effect but significantly attenuated the reduction of ALT level in the CCl_4_+iPS group at 24 h after CCl_4_ injury (n = 6, *p<0.05 vs. CCl_4_ group, ^#^ *p*<0.05 vs. CCl_4_+iPS group). (**D**) In CCl_4_-injured mice received iPS transfusion, the hepatocyte proliferation at the portal region at 48 h after CCl_4_ injury was significantly reduced by anti-IP-10 antibody. (**E**) Survival curve of mice treated with CCl_4,_ CCl_4_+iPS or rIP-10. All the mice were challenged with CCl_4_ at time 0, 24 and 48 h (n = 32). At 4 h after initial injury, half of the repetitive injured mice were randomized into two groups to receive iPS (n = 8) or rIP-10 (n = 8) treatment. The survivals of each group were observed until 72 h. Both rIP-10 and IPS treated groups had significant higher survival rates (*p<0.05, n = 6 in each group).

From above results, we showed that IP-10 could be an important hepatoprotective mediator. We then investigated whether or not recombinant IP-10 (rIP-10) can promote the proliferation of injured hepatocytes. The *in vitro* study showed that 0.5 or 5 ng of rIP-10 sufficiently increased the viability of injured hepatocytes at CCl_4_ concentration of 1.0 to 2.5 mM (Figure 5A). In injured mice, injection of rIP-10 significantly reduced the degree of liver damage and the effects of rIP-10 were compatible to iPS alone (Fig. 5B). Combined treatment of rIP-10 and iPS had no additive beneficial effects in injured mice. The application of anti-IP-10 neutralizing antibody attenuated the protective effects of iPS (Fig. 5C). In addition, the Ki67 or BrdU staining revealed that the proliferation of hepatocytes at portal regions after iPS infusion was significantly reduced by the anti-IP-10 neutralizing antibody (Fig. 5D).

### IPS Improved the Survival of Repetitive Injured Mice

To evaluate the survival effects of iPS and IP-10, the 72-hour survival rate was evaluated in repetitive CCl_4_-injured mice, to which two additional doses of CCl_4_ (given at 24 and 48 hours) were given after the first dose. Half of the repetitive injured mice were randomized into two groups to receive either iPS, or rIP-10 (5 ng) treatment. Both rIP-10 and IPS groups had significantly higher 72-hour survival rates (100% and 85.7%, respectively) when compared to the untreated group (53.3%, P<0.05) (Fig. 5E). No significant difference was noted between iPS and rIP-10 groups.

## Discussion

Acute massive or chronic persistent liver injuries can lead to liver failure. Developing a cell-based treatment or alternative therapeutic stratagem to reduce damage, prevent progression, and restore liver function is of important clinical relevance. This study demonstrated that the intravenously administered iPS reduced the intensity of injury and promoted hepatocyte proliferation. The transplanted iPS secreted IP-10 and help to increase hepatic IP-10 levels. The protective effect of iPS was attenuated by anti-IP-10 neutralizing antibody. In addition, applying rIP-10 protected hepatocytes and mice from CCl_4_ injury and improved their survival. These results demonstrated that iPS transplantation facilitated liver damage repair and promoted hepatocyte regeneration in order to restore liver function. Hepatic IP-10 was an important factor that mediated the beneficial effect of iPS in acute liver injury.

Because iPS have the potential to proliferate indefinitely and differentiated into different cell types, hepatocytes generated from iPS can be a valuable alternative source of primary hepatocytes [7], [12]. However, it is unknown if the hepatocytes derived from iPS can provide adequate function better than iPS in the recipients. To answer this question, we compared the therapeutic effects of iPS and iHL. It was found that both iPS and iHL reduced serum ALT and AST levels, however, the injury areas were not synchronously reduced by iHL. Moreover, iHL promoted less hepatocytes proliferation than iPS did. The actual causes of the functional and histological discordance of iHL are unclear. But the same presentation has ever been observed in cell transplantation using mesenchymal stem cells-derived hepatocytes [25]. One possible factor that may account for this discrepancy could be that differentiation in the *in vitro* culture conditions were unlike the native *in vivo* environment. Even though the iHL have displayed characteristic functions of primary hepatocyte, the *in vitro* differentiated iHL might have lost some of the potency of the iPS to resist injury and to promote repopulation of liver parenchyma cells. Kuo et al. had found a similar result that the mesenchymal stem cells-derived hepatocytes did not offer better functionality than the undifferentiated mesenchymal stem cells [17]. Another factor might be the limited success rate of hepatic engraftment after cell transplantation. We found that the amount of iHL localized in the damaged liver was much less than that of iPS. It is possible that there were not enough numbers of engrafted iHL to produce similar protective effects as iPS. The critical contribution of cell engraftment has been suggested in a study that chimeric mice whose entire liver engrafted with iPS cell-derived hepatocytes recovered from liver failure rapidly [11]. Therefore, whether the iPS cell-derived hepatocytes act as a better cellular source for transplantation required further investigation.

Cytokines and chemokines are important mediators of immune and inflammatory responses. We found two chemokines, IP-10 (or CXCL10) and MIG (or CXCL9), increased prominently in the injured liver after iPS infusion. They are two related chemokines belonging to the CXC subfamily [26]. Both have similar activities and share a common CXCR3 receptor. Increased expression of IP-10 has been found in chronic hepatitis [19], [27], [28], while MIG was associated with liver fibrosis [29]. In the current study, CCl_4_ injury increased the expression of IP-10 and MIG. The infusion of iPS further increased their expression. The increased expression of IP-10 and MIG could be caused directly by iPS or indirectly by their inducers such as IFN-γ [26] and TNF-α [30]. Marked increase of IP-10 secretion has been observed in endothelial cells co-stimulated by IFN-γ and TNF-α [28]. A recent study shows that type I IFN (IFN α/β) is required for IP-10 production in ischemia/reperfusion liver injury [24]. In our current study, the expression of IFN-α and IFN-γ expressions in the injured liver were low and were not affected by IPS. Moreover, the level of TNF-α mRNA was reduced by iPS. These results implied that the increases of IP-10 and MIG were less likely to be induced by IFN or TNF-α. Thus, the results here demonstrated that iPS transfusion could increase IP-10 in the injured liver.

The roles of CXCR3-related chemokines in tissue damage have been studied in various types of injury and in different organs system. The results are controversial. IP-10 inhibits bleomycin-induced pulmonary fibrosis [31], [32], while blockade of IP-10 attenuates chronic colitis and promotes renal fibrosis [33], [34]. In the liver, IP-10 is protective in hapten-induced hepatitis and acetaminophen-induced liver injury [21], [22]. It has been proposed to mediating not only hepatic inflammatory response but also liver regeneration in multiple models of hepatic and bile duct injury [30]. However, IP-10 may not be beneficial in certain conditions. It was reported that knock out IP-10 protected mice from ischemia/reperfusion liver injury [24]. Yoneyam et al. demonstrated that neutralization of IP-10 could accelerate liver regeneration and rIP-10 (100 and 1000 ng/ml) inhibited *in vitro* HepG2 proliferation [35]. In the present study, we found that the iPS-induced hepatic IP-10 was protective and rIP-10 (0.5 and 5 ng/ml) may promote *in vitro* AML12 proliferation, but at lower doses. The differential effects of IP-10 on the proliferative responses of hepatocytes could be related to dose, different cell types or other yet unidentified factors. As proposed in human hepatitis C infection, chemokines are crucial for viral elimination but inappropriate expression can drive inflammation and tissue damage [36]. To realize the complex regulatory mechanism of IP-10, it required more investigations in the future.

We did not observe teratoma formation in our mice for 6 months (Fig. S3). However, to minimize the risk of tumor growth, it stands a reason to characterize if IP-10 is responsible for the effect of iPS. Thus, IP-10 may potentially replace iPS or help reduce the cell numbers of iPS used. In the current study, we found that rIP-10 could exert proliferative and protective effects on healthy and injured hepatocytes. In addition, neutralizing the effects of IP-10 resulted in greater liver injury and an obvious decrease of proliferating hepatocytes. To identify the cellular sources of IP-10, we demonstrated that both iPS and hepatocytes could release small amount of iP-10 *in vitro*. Importantly, the expression of IP-10 by hepatocytes in injured liver treated with iPS increased more than 5 fold than those without iPS treatment. These results implicated that iPS contributed to the increased expression of hepatic IP-10 by hepatocytes in the injured liver. It is possible that the secreted IP-10 could subsequently act like an autocrine or paracrine agent on adjacent viable hepatocytes to exert its protective effects. In the survival analysis, about half of the mice died from repetitive CCl_4_ injuries within 72 hours while treatments of iPS or rIP-10 effectively reduced their mortality. Collectively, our study results implicated that by the help of IP-10, iPS alleviated the intensity of injury and promoted hepatocytes to leave their growth-arrested state and become mitotically active to repopulate and restore the function of the acute injured liver. However, there are other unrevealed mechanisms responsible for the beneficial effect of iPS. Further studies are needed to clarify the exact interactions among iPS, IP-10 and hepatocytes *in vivo* in the injured liver.

In conclusion, our results demonstrated that iPS transfusion reduced serum ALT, AST and the areas of necrosis in acute CCl_4_-injured liver. The treatment of iPS enhances the expression of hepatic IP-10, which is an important hepatoprotective mediator to facilitate hepatocyte regeneration, restoration of liver function, and improve survival in the acute CCl_4_-injured liver.

## Materials and Methods

### Experimental Design and Animal Study

Mice (C57/B6, 8 to 10 weeks) were housed in cage and were allowed free access to food and water. Mouse was given carbon tetrachloride (CCl_4_, Sigma) in mineral oil (0.35 µl/g, single dose, i.p.) to induce liver injury. At 4 h post-injury, mice were randomized to receive vehicle (PBS), iPS or iHL (2×10^6^ cells/in 100 µl PBS) infusions via tail veins. At given time point, about 100 µl of mice blood were drawn from facial veins for liver biochemistry. When mice were sacrificed, blood were drawn from the heart and the liver was harvested and prepared for subsequent experiments including histochemistry, cytokine assay, protein and gene expression analysis. For flow cytometry and primary liver cells studies, total liver cells were isolated at 24 h post-injury from normal and the CCl_4_-injured mice received vehicle or iPS transfusion. For neutralizing antibody study, mice were given two doses (0.5 µg/dose, i.p.) of anti-IP-10 antibodies (Abcam, ab9938, Cambridge, UK) at 4 h before and 4 h after injury. For 72 h survival study, mice received repetitive CCl_4_ injury at 0, 24 and 48 h. The iPS (2×10^6^ cells/in 100 µl PBS) or recombinant IP-10 (rIP-10, 0.5 ng, PreproTech, Rocky Hill, NJ, USA) were given once at 4 h and the mortality rate of mice was observed until 72 h post-injury. All animals received humane care according to the Guide for the Care and Use of Laboratory Animals prepared by the National Academy of Sciences (NIH publication no. 86–23, revised 1985) and approved by the Institutional Animal Care and Use Committee (IACUC) of Taipei Veterans General Hospital (VGH99-173). All experiments adhered to the American Physiological Society Guiding Principles for the Care and Use of Laboratory Animals.

### Cell Cultures Studies

Mouse germline-competent iPS were provided by Kyoto University (Dr. Shinya Yamanaka) and RIKEN BRC, Japan [8]. IPS were cultured as previously described [9]. The iPS were successfully induced to differentiate into hepatocyte-like cells with functions resembling primary hepatocytes (Supplementary Methods and Results S1, Fig. S1–S2). Mouse none-transformed hepatocyte cell line, AML12 (ATCC CRL-2254), was grown in 10% DMEM. In co-culture experiment, hepatocytes (3×10^4^ cells) were placed on the bottom. CCl_4_ at concentration of 2.0 mM was used to induce approximately 50% death of hepatocytes after 24 h. The iPS placed on the cell-culture inserts (0.4 µm, Transwell) at density of 1%, 3% or 10% of hepatocyte’s numbers were transferred at 4 h post-injury and co-incubated until 24 h. For rIP-10 study, AML12 hepatocytes were seeded on 24-well plates at the same density. The rIP-10 (0.5 ng or 5 ng/ml) was given at 4 h post-injury. The viability of AML12 hepatocytes was evaluated at 24 h by methyl thiazol tetrazolium (MTT, Sigma) assay [37].

### Histological Quantification of Liver Injury

The paraffin sections of livers were stained by hematoxylin-eosin (H.E) stain and photo-taken under microscopy at 40× magnification to evaluate the degree of injury. Necrotic area were determined by measuring five independent fields per liver using a computerized morphometry system (MicroCam, M&T OPTICS, Taiwan) and expressed as percentage of the filed area.

### Detection of Proliferating Hepatocytes

At 2 h prior to sacrifice, mice were injected with 5-bromo-2′-deoxyuridine (BrdU, 50 mg/kg, i.p., Sigma). The peroxidase-coupled mouse monoclonal anti-BrdU (DAKO, M0744) and anti-Ki67 (DAKO, M7249) were used in subsequent immunohistochemistry study for detecting proliferative hepatocytes. Ten pictures of the interested areas (different portal and central vein areas) per animal were photo-taken under microscopy at x200 magnification. The mean numbers of BrdU-positive or Ki67-positive cells of per area per animal were used in statistical analysis.

### Fluorescence Cell Labeling

In serum-free medium, 1×10^6^ mouse iPS were incubated with 1,1′-dioctadecyl-3,3,3′,3′-tetramethylindocarbocyanine perchlorate, (10 µM, Vybrant® DiI, Molecular Probes, Eugene, OR) for 15 minutes and centrifuged at 1500 rpm for 5 minutes at 37°C. The supernatant was later removed and the cells were re-suspended in PBS for experiment. The labeling efficiency of DiI on iPS was >99% using flow cytometry.

### Preparations of Total Liver Cells and Flow Cytometry Studies

Mouse total liver cells, i.e. the primary hepatocytes (HC) and nonparenchymal cells (Npc), were prepared by collagenase perfusion and isodensity gradient centrifugation. Briefly, under anesthesia the portal vein was inserted by 27-gauge catheter and the inferior vena cava was cut. The liver was perfused by collagenase digestion buffer, which is Ca2^+^, Mg2^+^-free Hank’s balanced salt solution (HBSS) containing collagenase type IV (3 mg/30 ml, Sigma) at 37°C. After perfusion, the digested liver was excised, dispersed and filtered through 100 µm cell strainers (BD Biosciences). The HC were separated from the Npc by sequential low speed centrifugation at 50 g. The viable HC and the Npc were further purified by the gradient centrifugation using Optiprep (Sigma) according to the manufacturer’s recommendation. For the flow cytometry study, total liver cells were isolated by the same perfusion technique. After perfusion, the liver was homogenized with collagenase digestion buffer and incubated at 37°C for 40 min under gentle agitation. The digested liver homogenate was re-suspended in single cell suspension for FACS analysis. The red blood cells were removed by ACK lysing buffer.

### RNA Extraction, and Reverse Transcription Polymerase Chain Reaction

Total RNA was isolated using TRIzol reagent (Sigma). One µg total RNA was reverse-transcribed to cDNA by MMLV high performance reverse transcriptase (Epicentre, WI) with random primers. The primers used were listed in table (Table S1). Quantitative real-time PCR was performed using Fast SYBR green PCR Master Mix according to the manufacturer’s instructions (7900HT, Applied Biosystems, CA).

### Western Blotting

Tissue lysates were prepared in a buffer containing 50 mM Tris-HCl, pH 7.4, 150 mM NaCl, 0.25% deoxychoic acid, 1% NP40, 1 mM EDTA, 1 mM Na orthovanadate, 1 mM Na fluoride, 1 mM phenylmethylsulfony fluoride, 1 ug/ml aprotinin, 1 ug/ml leupeptin and 1 ug/ml peptstain, on ice as described before [37]. The concentrations of sample proteins were determined using the Protein Assay kit (Bio-Rad, Hercules, CA). Specific amounts of total protein were subjected to 10% SDS–PAGE gel electrophoresis and then transferred to PVDF membranes. Membranes were blocked with 5% non-fat milk and incubated overnight at 4°C with primary antibodies. The membranes were then washed in Tris-buffered saline Tween-20 (TBST) for 5 times and then incubated with horseradish peroxidase-conjugated secondary antibody for 2 h at room temperature. The membrane was then washed for six times by TBST and specific bands were visualized by ECL (Pierce Biotechnology, Rockford, IL) and captured with a digital image system (ChemiGenius2 photo-documentation system, Syngenes, Cambridge, UK).

### Cytokine Array and IP-10 ELISA

The liver tissues of the CCl_4_-injured mice without or with iPS treatment were homogenized and prepared in PBS with protease inhibitors (10 µg/mL Aprotinin, 10 µg/mL Leupeptin, and 10 µg/mL Pepstatin) and 1% Triton X-100. The tissue lysates were centrifuged at 10,000 g for 5 minutes to remove cell debris. The protein concentrations were quantified (DC-Bradford protein assay, Bradford, Bio-Rad, Hercules, CA, USA) and 200 µg of proteins were used for the analysis of cytokines by the commercialized assay kits (Mouse cytokine array panel A and IP-10 Immunoassay, R&D, MN) according the manufacture’s instruction. The expression of individual cytokines in injured liver received iPS treatment was quantified by densitometry and expressed as fold change relative to their expressions in the injured liver without iPS treatment.

### Statistical Analysis

The results were expressed as mean±SEM. Statistical analysis was performed by using an independent Student t test and one- and two-way ANOVA with Tukey post hoc test when appropriate. The survival analysis was performed by using logrank test. A *p *value <0.05 was considered statistically significant.

## Supporting Information

Figure S1 **Characterization of hepatocyte differentiation potential in induced pluripotent stem (iPS) cells. (A)** Morphology of the iPS cells on feeder layer of fibroblasts and **(B)** iPS-derived hepatocyte-like (iHL) cells after hepatogenic induction. Insert picture is normal hepatocyte. **(C–E)** Hepatocyte-specific protein markers expressed in iHL cells. The hepatic specific markers AFP, ALB and HNF-3β were detected by immunofluorescence assay. (F) Hepatocyte-specific transcripts expressed in iHL cells RNA from adult liver cells (lane 1) and fetal liver cells (lane 2) represent the positive control while RNA from mouse embryonic fibroblasts (MEF, lane 3) represent the negative control. AFP, α-fetal protein; ALB, albumin; HNF-3β, hepatocyte nuclear factor-3β; TTR, Transthyretin; AAT, α-antitrypsin; TAT, tyrosine-aminotransferase; G-6-P glucose-6-phosphatase.
(DOC)

Figure S2 **Functional characterization and immunofluorescence (IF) staining of induced pluripotent stem (iPS) cell-derived hepatocyte-like cells.** (**A**) Phase contrast and IF images showed DiI-Ac-LDL uptake by differentiated iPS cell after two weeks hepatogenic induction. (**B**) Positive PAS stain for glycogen storage in iPS cell-derived hepatocytes. (**C**) IF stain showed that 9B2 antigens (red) were expressed at the junction between adjacent hepatocytes. F-actin (green) and DAPI (blue).
(DOC)

Figure S3 **The 6-month teratoma observation study.** The iPS cells were labeled with GFP (iPSC-GFP) then injected into mice in our experimental system (N = 4). The total follow up time was 6 months. The iPSC-GFP positive signals were examined by the Ex vivo GFP imaging. The results demonstrated that there were no GFP signal could be found by Ex vivo GFP imaging. In addition, no tumor detected by histological when detail survey were performed in multiple organs including liver, lung, stomach, intestine, colon, kidney, bladder, and brain.
(DOC)

Figure S4 **Interferons (IFN) and TNF-α are not inducers of IP-10.** **(A)** In the injured liver, the expression of IFN-γ and IFN-α mRNA were reduced and remained low despite iPS infusion. There was no significant difference in IFN-λ. **(B)** Hepatic TNF-α increased after injury but was reduced by iPS infusion. The TNF-α receptor type 1 (TNF-α R1) expression increased significantly after injury. IPS infusion did not alter the expression levels of TNF-α R1 mRNA (n = 6, *p<0.05 vs. normal control, ^#^P<0.05, vs. CCl4)
(DOC)

Supplementary Methods and Results S1 (DOC)

Table S1 **Primer sequences used in real time-PCR.**
(DOC)

Table S2 **Organ distribution of iPS injected into CCl_4_-injured mice.**
(DOC)
