# Supplementary figures and images for: The Role of Interferon-γ Inducible Protein-10 in a Mouse Model of Acute Liver Injury Post Induced Pluripotent Stem Cells Transplantation
Source: PLoS One. 2012 Dec 5;7(12):e50577. doi: 10.1371/journal.pone.0050577 (PMC3515611; doi:10.1371/journal.pone.0050577)

**Figure S1.**


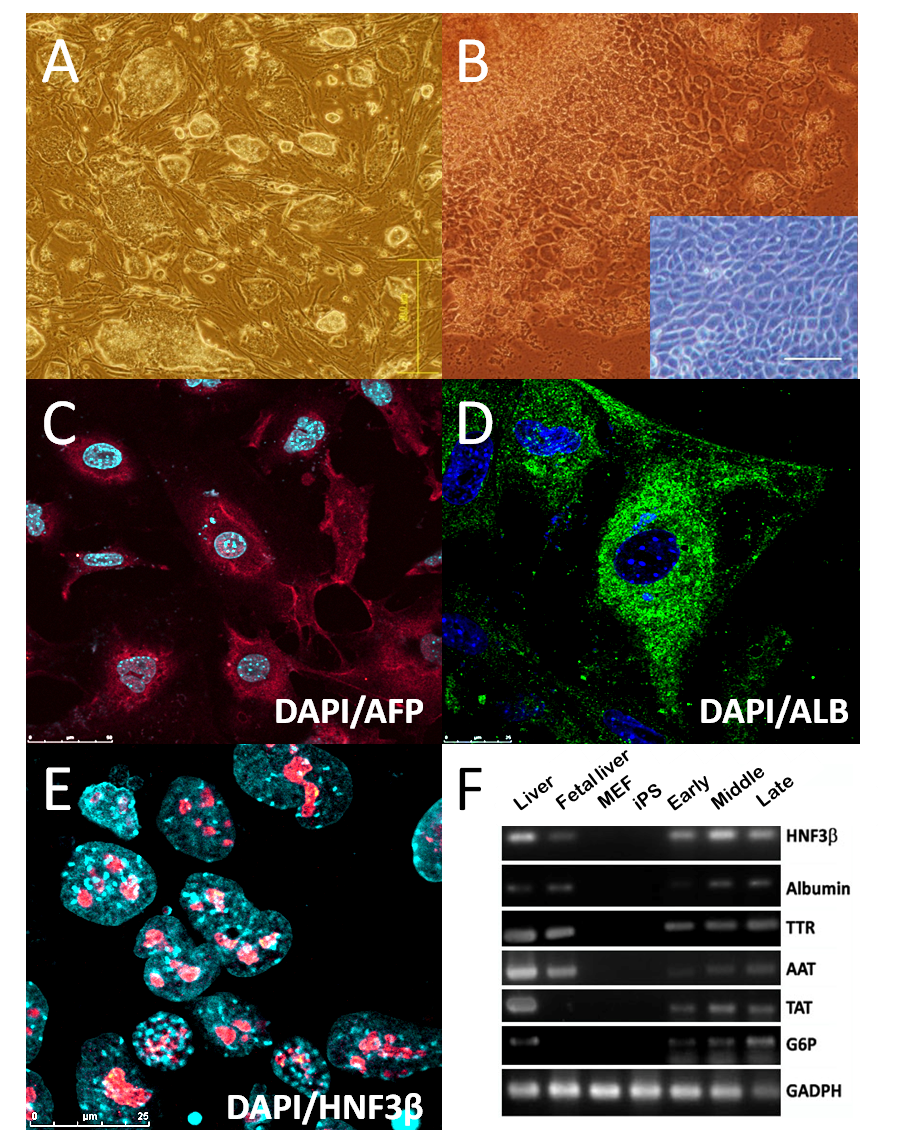

Supplement: Figure S1 — Characterization of hepatocyte differentiation potential in induced pluripotent stem (iPS) cells. (A) Morphology of the iPS cells on feeder layer of fibroblasts and (B) iPS-derived hepatocyte-like (iHL) cells after hepatogenic induction. Insert picture is normal hepatocyte. (C–E) Hepatocyte-specific protein markers expressed in iHL cells. The hepatic specific markers AFP, ALB and HNF-3β were detected by immunofluorescence assay. (F) Hepatocyte-specific transcripts expressed in iHL cells RNA from adult liver cells (lane 1) and fetal liver cells (lane 2) represent the positive control while RNA from mouse embryonic fibroblasts (MEF, lane 3) represent the negative control. AFP, α-fetal protein; ALB, albumin; HNF-3β, hepatocyte nuclear factor-3β; TTR, Transthyretin; AAT, α-antitrypsin; TAT, tyrosine-aminotransferase; G-6-P glucose-6-phosphatase. (DOC) [file pone.0050577.s001.doc]

**Figure S2**


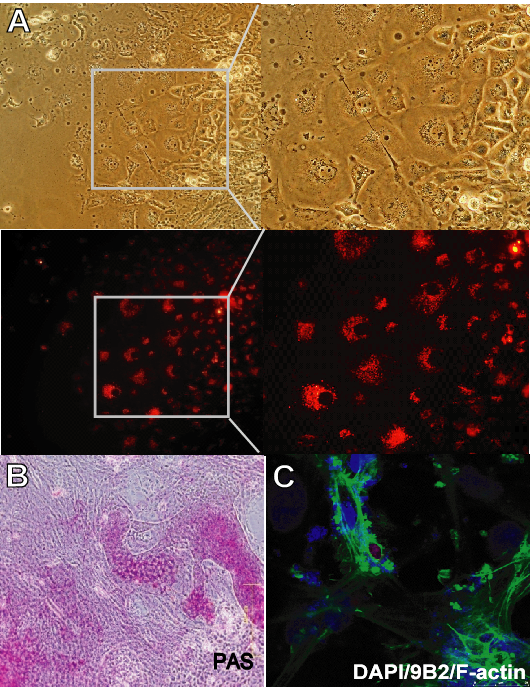

Supplement: Figure S2 — Functional characterization and immunofluorescence (IF) staining of induced pluripotent stem (iPS) cell-derived hepatocyte-like cells. (A) Phase contrast and IF images showed DiI-Ac-LDL uptake by differentiated iPS cell after two weeks hepatogenic induction. (B) Positive PAS stain for glycogen storage in iPS cell-derived hepatocytes. (C) IF stain showed that 9B2 antigens (red) were expressed at the junction between adjacent hepatocytes. F-actin (green) and DAPI (blue). (DOC) [file pone.0050577.s002.doc]

**Figure S3.**


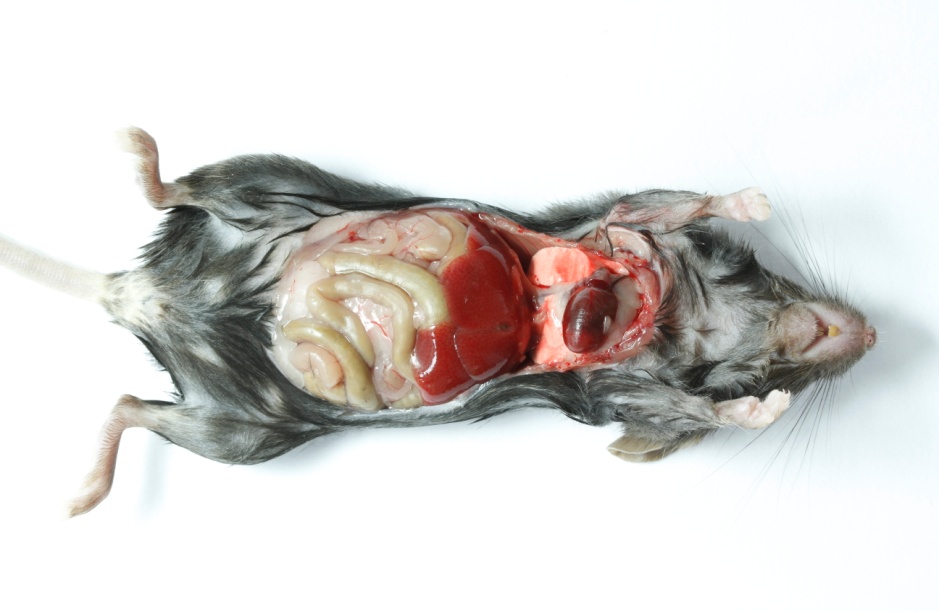

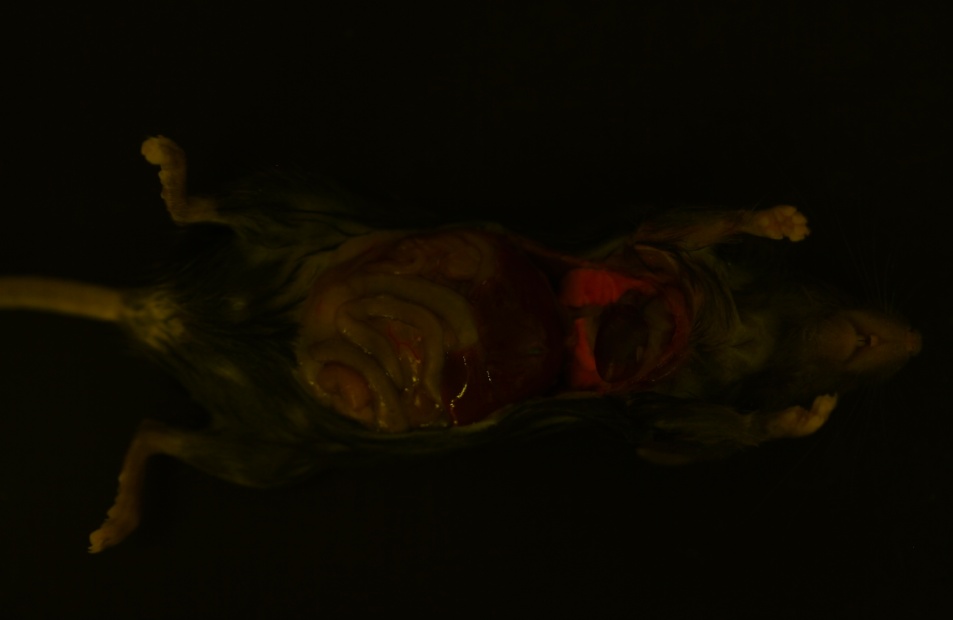

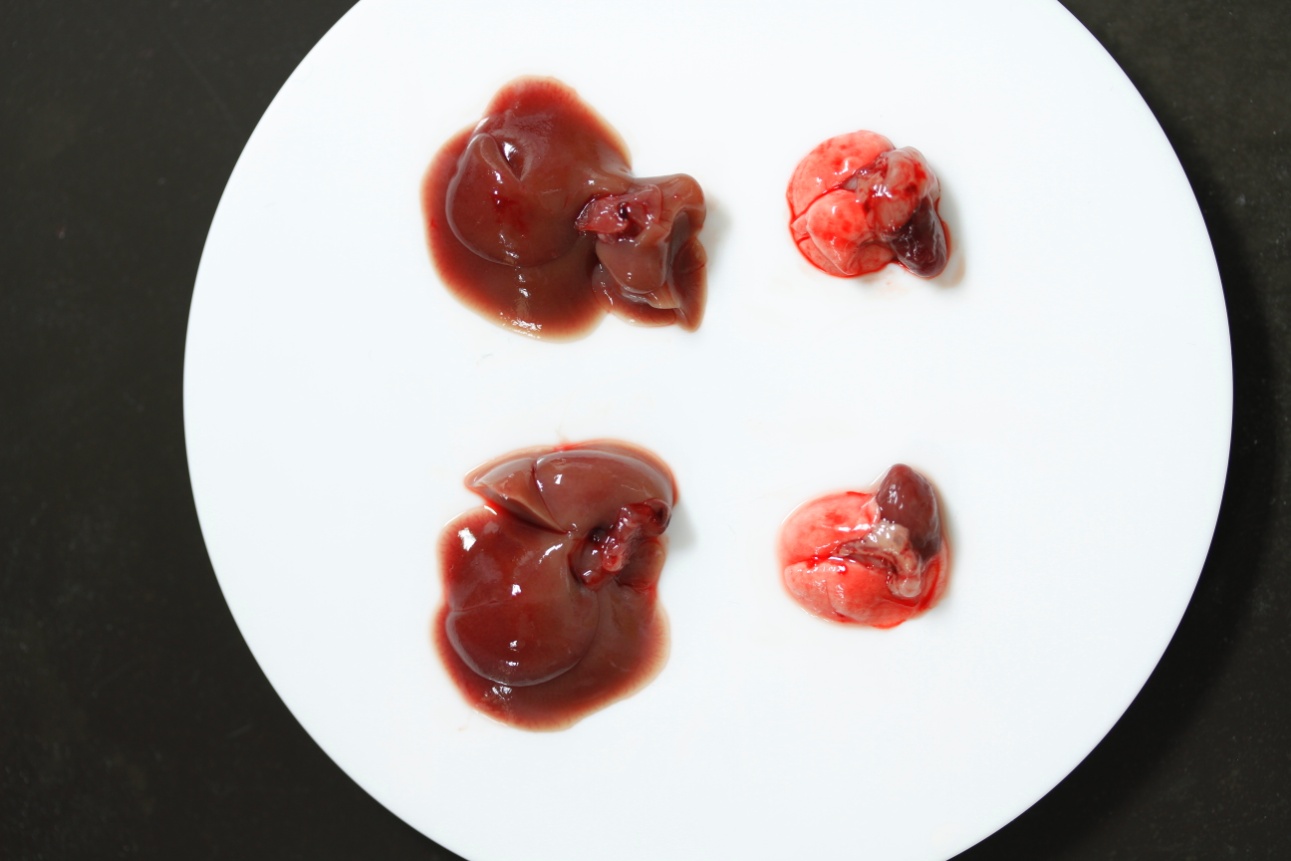

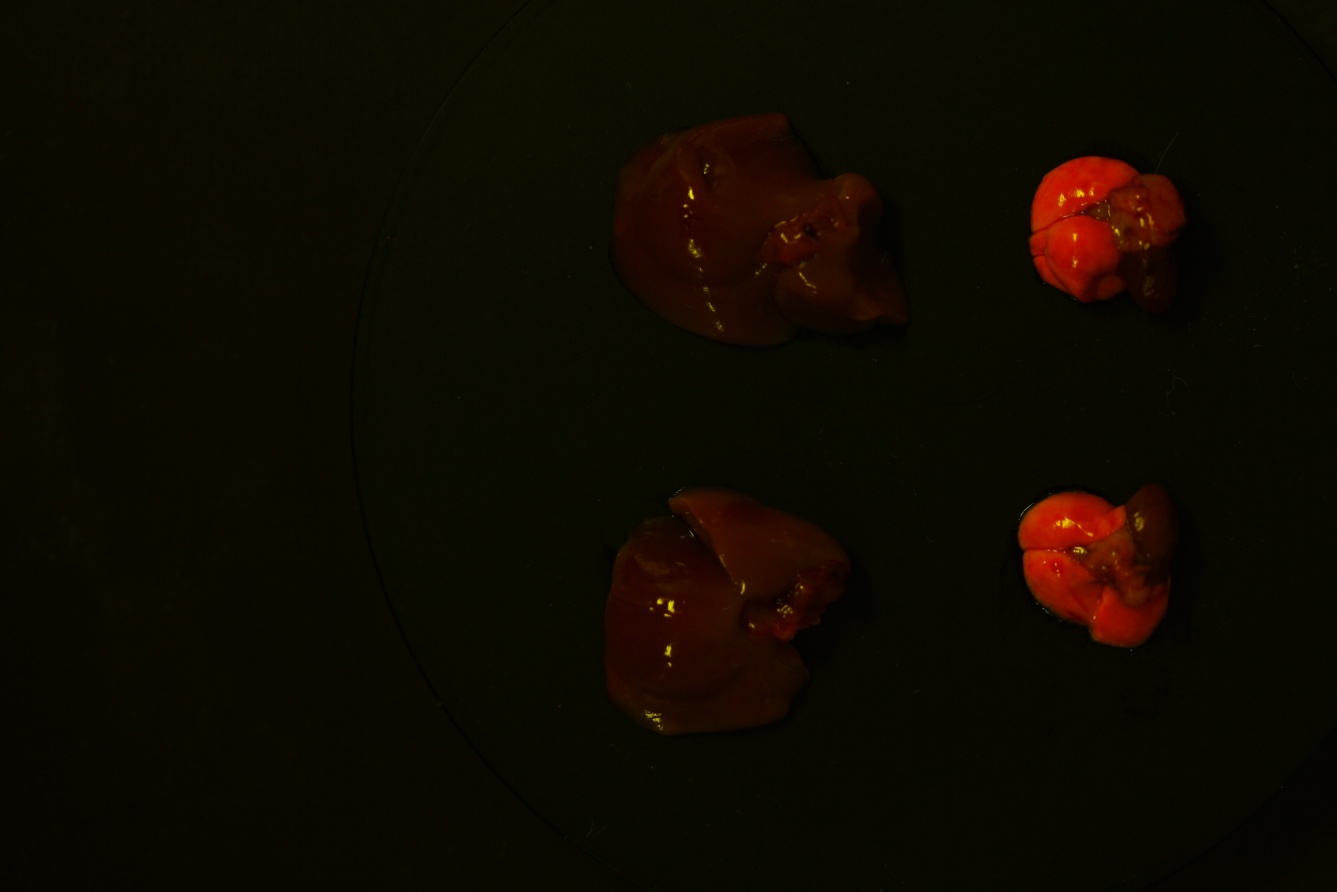


**GFP Imaging**

**GFP Imaging**

Supplement: Figure S3 — The 6-month teratoma observation study. The iPS cells were labeled with GFP (iPSC-GFP) then injected into mice in our experimental system (N = 4). The total follow up time was 6 months. The iPSC-GFP positive signals were examined by the Ex vivo GFP imaging. The results demonstrated that there were no GFP signal could be found by Ex vivo GFP imaging. In addition, no tumor detected by histological when detail survey were performed in multiple organs including liver, lung, stomach, intestine, colon, kidney, bladder, and brain. (DOC) [file pone.0050577.s003.doc]
