# Supplementary material for: The Role of Interferon-γ Inducible Protein-10 in a Mouse Model of Acute Liver Injury Post Induced Pluripotent Stem Cells Transplantation
Source: PLoS One. 2012 Dec 5;7(12):e50577. doi: 10.1371/journal.pone.0050577 (PMC3515611; doi:10.1371/journal.pone.0050577)
Supplement: Figure S4 — Interferons (IFN) and TNF-α are not inducers of IP-10. (A) In the injured liver, the expression of IFN-γ and IFN-α mRNA were reduced and remained low despite iPS infusion. There was no significant difference in IFN-λ. (B) Hepatic TNF-α increased after injury but was reduced by iPS infusion. The TNF-α receptor type 1 (TNF-α R1) expression increased significantly after injury. IPS infusion did not alter the expression levels of TNF-α R1 mRNA (n = 6, *p<0.05 vs. normal control, #P<0.05, vs. CCl4) (DOC) [file pone.0050577.s004.doc]

**Figure S4**

**A**

.

B
