# Supplementary material for: The Role of Interferon-γ Inducible Protein-10 in a Mouse Model of Acute Liver Injury Post Induced Pluripotent Stem Cells Transplantation
Source: PLoS One. 2012 Dec 5;7(12):e50577. doi: 10.1371/journal.pone.0050577 (PMC3515611; doi:10.1371/journal.pone.0050577)
Supplement: Supplementary Methods and Results S1 — (DOC) [file pone.0050577.s005.doc]

**Supplementary Methods and Results**

**Methods**

*Growth Factors and Antibodies*

Basic FGF (bFGF) and hepatocyte growth factor (HGF) were purchased from PeproTech (Rocky Hill, NJ). Nicotinamide, dexamethasone (Dex), insulin- transferrin-selenium (ITS) and Triton X-100 were obtained from Sigma-Aldrich. TRIzol Reagent was obtained from Invitrogen. The anti-HNF-3b and anti-α-fetoprotein (AFP) antibodies were purchased from Santa Cruz biotechnology while the anti-albumin antibody was obtained from Millipore.

*Culture and hepatogenic differentiation of iPS cells*

Mouse germline-competent iPS cells were provided by Kyoto University (Dr. Shinya Yamanaka) and RIKEN BRC, Japan . IPS cells were cultured as previously described . At the same time, the iPS cells were differentiated into hepatocytes by the 2-step procedure. In brief, the iPS cells were seeded at 2x10^4^ cells/cm^2^ maintained in DMEM containing 10% fetal bovine serum, 100 U/ml penicillin and 10 μg/ml streptomycin in gelatin-coated plates, prior to a 2-step procedure. Colonies were detached from culture plates by using trypsin (0.25%, Invitrogen) at room temperature for 10 minutes and placed in ultralow attachment plates in suspension culture to from embryonic bodies (EBs) for 4 days. EBs were placed on 0.1% gelatin-coated plates and then differentiated by using step-1 differentiating medium, consisting of DMEM supplemented with 20 ng/ml HGF, 10 ng/ml bFGF and 0.61 g/L nicotinamide. After 7 days, step-1 differentiating medium was changed to step-2 maturation medium containing DMEM supplemented with 0.1 μM nicotinamide, dexamethason (Sigma) and 1% insulin-transferrin-selenium (ITS; Sigma). Medium changes were performed twice per week. The iPS cells treated with medium supplemented with no growth factors were used as the negative control.

*Periodic Acid-Schiff (PAS) Stain for Glycogen*

Cells were ﬁxed in 4% paraformaldehyde, and then permeabilized with 0.1% Triton X-100 for 10 minutes. Samples were then oxidized in 1% periodic acid for 5 minutes, rinsed 3 times in deionized (d) H_2_O, treated with Schiff’s reagent for 15 minutes, and rinsed in (d)H_2_O for 5-10 minutes. Samples were counterstained with Mayer’s hematoxylin for 1 minute and then rinsed in (d)H2O. Samples were observed under light microscope.

*Cellular Uptake assay of Low-Density Lipoprotein (LDL)*

Uptake capability of 1,1’-dioctadecyl-1-,3,3,3’,3’,-tetramethyl-indo-carbocyanine perchlorate conjugated to acetylated-LDL (DiI-Ac-LDL, AbD Serotec) of iPS cells and differentiated cells was determined by fluorescent microscopy. Cells were incubated with 20 mg/ml DiI-AC-LDL at 37°C for 24 h. Incorporation of DiI-Ac-LDL into cells was visualized by fluorescence microscopy.

**Results**

***Characterizing the potential of hepatocyte differentiation in iPS***

IPS was cultured on inactivated mouse embryonic fibroblasts (MEF) (Fig. S1A). Under the induction of hepatogenic medium, iPS developed broadened and cuboidal morphology (Fig. S1B). These differentiated cells were referred as iPS-derived hepatocyte-like (iHL) cells. The iHL at 7, 14 and 21 days post-induction expressed hepatocyte-specific markers HNF-3b, albumin, transthyretin, α-antitrypsin, tyrosine-aminotransferase, and glucose-6-phosphatase (Figs. S1C-S1E). In contrast, the iPS and MEF cells did not express any hepatocyte-specific markers (Fig. S1F). Functional evaluation demonstrated that iHL cells exhibited the abilities to uptake LDL and store glycogen (Fig. S2). The iHL cells after 14 days of induction were used for experiments.

**References**

1. Takahashi K, Yamanaka S (2006) Induction of pluripotent stem cells from mouse embryonic and adult fibroblast cultures by defined factors. Cell 126: 663-676.

2. Okita K, Ichisaka T, Yamanaka S (2007) Generation of germline-competent induced pluripotent stem cells. Nature 448: 313-317.

3. Kao CL, Tai LK, Chiou SH, Chen YJ, Lee KH, et al. (2010) Resveratrol promotes osteogenic differentiation and protects against dexamethasone damage in murine induced pluripotent stem cells. Stem Cells Dev 19: 247-258.

**Table S1.** Primer sequences used in real time-PCR

| Name | Sequences | | Length ID | |
| --- | --- | --- | --- | --- |
| IP-10 | | CGTCATTTTCTGCCTCATCCT  TGGTCTTAGATTCCGGATTCAG | | 227 NM_021274 |
| MIG | | ACTCAGCTCTGCCATGAAGTCCGC  AAAGGCTGCTCTGCCAGGGAAGGC | | 479 NM_008599 |
| ITAC | | ATGAACGGCTGCGACAAAGT  GCATGTTCCAAGACAGCAGA | | 225 NM_019494 |
| CXCR3 | | AGAATCATCCTGGTCTGAGACA  AAAGATAGGGCATGGCAGCTA | | 256 NM_009910 |
| IFN-α | | CCTGTGTGATGCAACAGGTC  TCACTCCTCCTTGCTCAATC | | 209 NM_010505 |
| IFN-γ | | TGATGGCCTGATTGTCTTTCAA  GGATATCTGGAGGAACTGGCAA | | 110 NM_0083337 |
| IFN-λ | | AGCTGCAGGCCTTCAAAAAG  TGGGAGTGAATGTGGCTCAG | | 244 NM_0010243673 |
| GAPDH | | TGTTGAAGTCACAGGAGACAACCT  AACCTGCCAAGTATGATGACATCA | | 111 XR_030913 |

**Table S2.** Organ distribution of iPS injected into CCl_4_-injured mice

| Location | iPS (-)  % (n=5) | iPS (+)  % (n=5) | *p value* |
| --- | --- | --- | --- |
| Liver | 0.00±0.00 | 2.66±0.46 | 0.012 |
| Spleen | 0.14±0.03 | 4.74±1.68 | 0.028 |
| Lung | 0.03±0.02 | 2.21±0.25 | 0.00002 |
| Bone Marrow | 0.11±0.06 | 1.82±0.56 | 0.0159 |

Induced pluripotent stem (iPS) cells were labeled with fluorescence dye DiI with >99% labeling efficiency. The background fluorescence of the injured liver that received no iPS cells was used as reference. The percentages of positive cells derived from five independent experiments were used for analysis.

**Figure S1.**


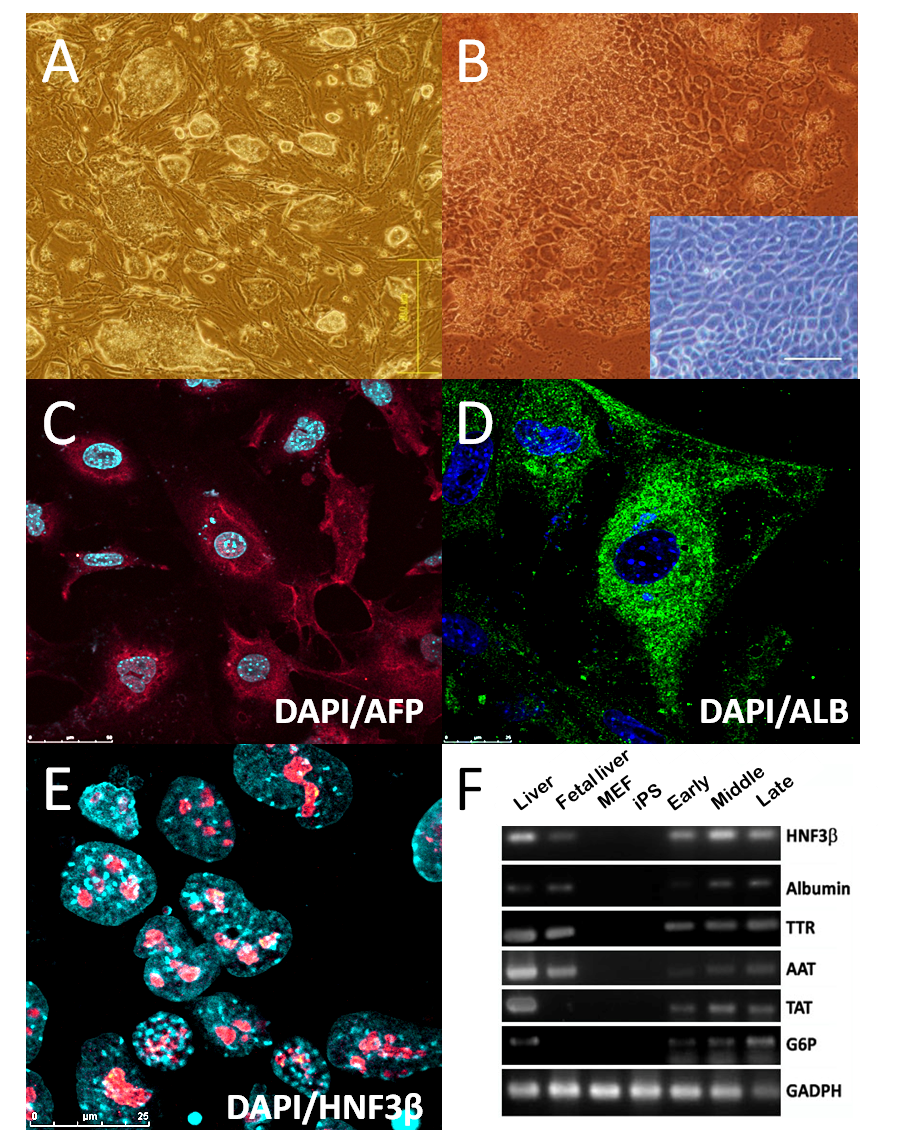


**Figure S2**


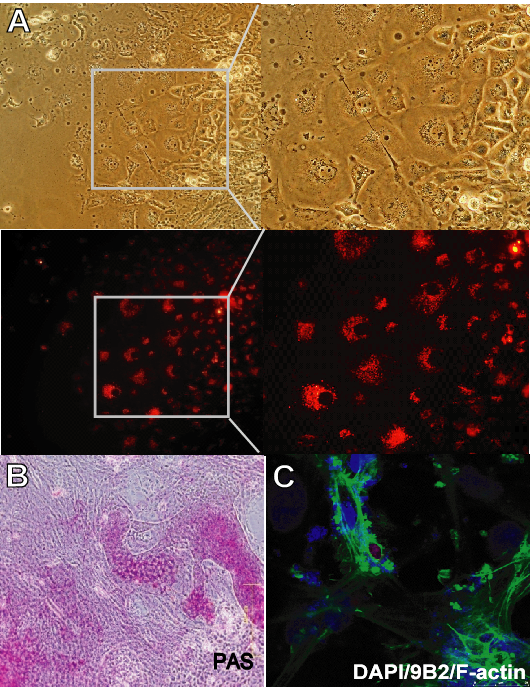


**Figure S3.**


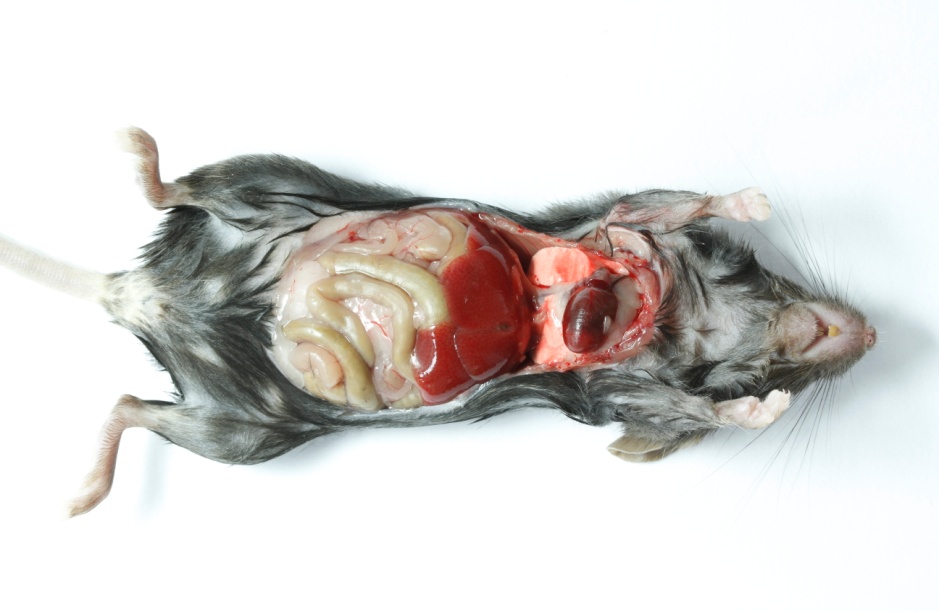

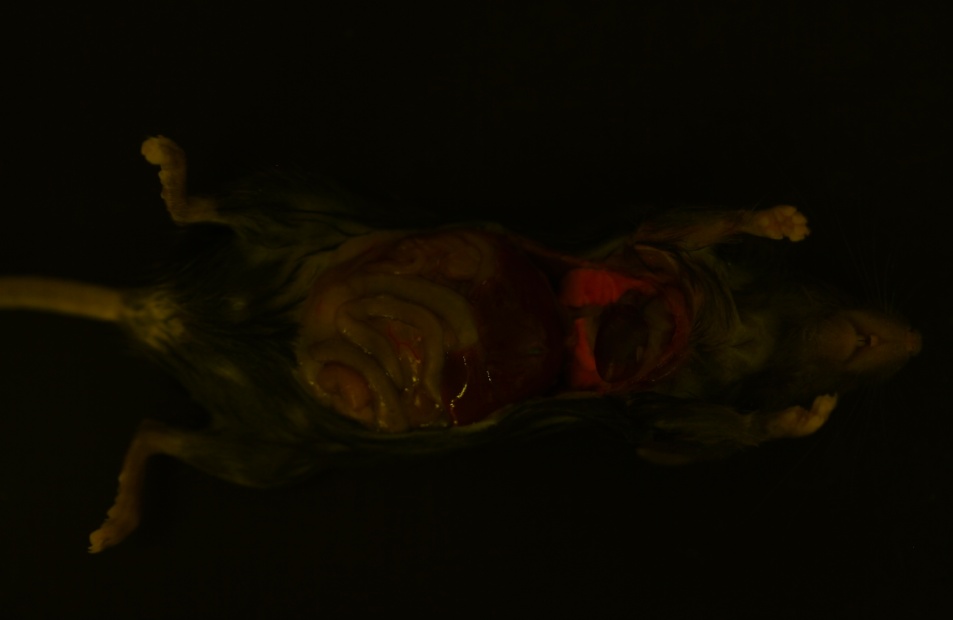

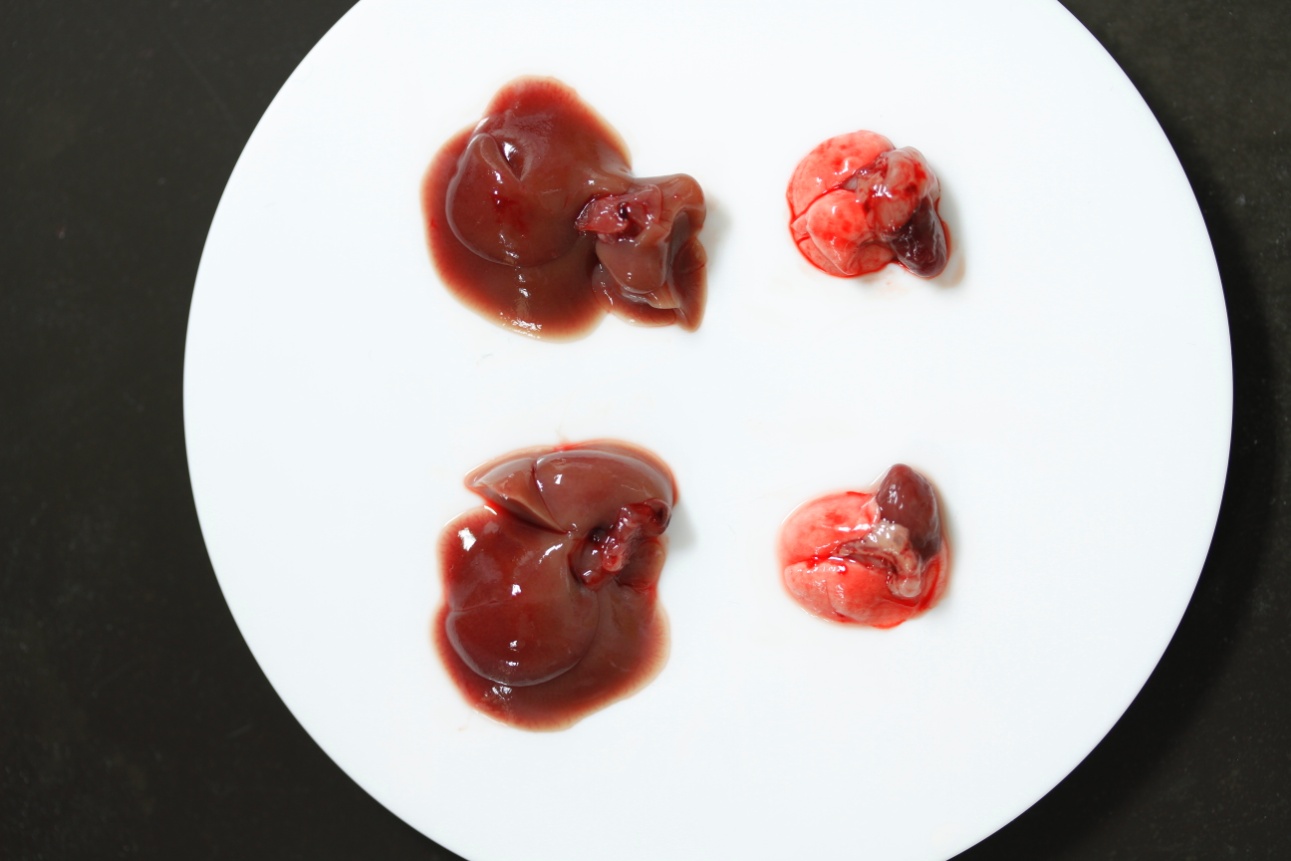

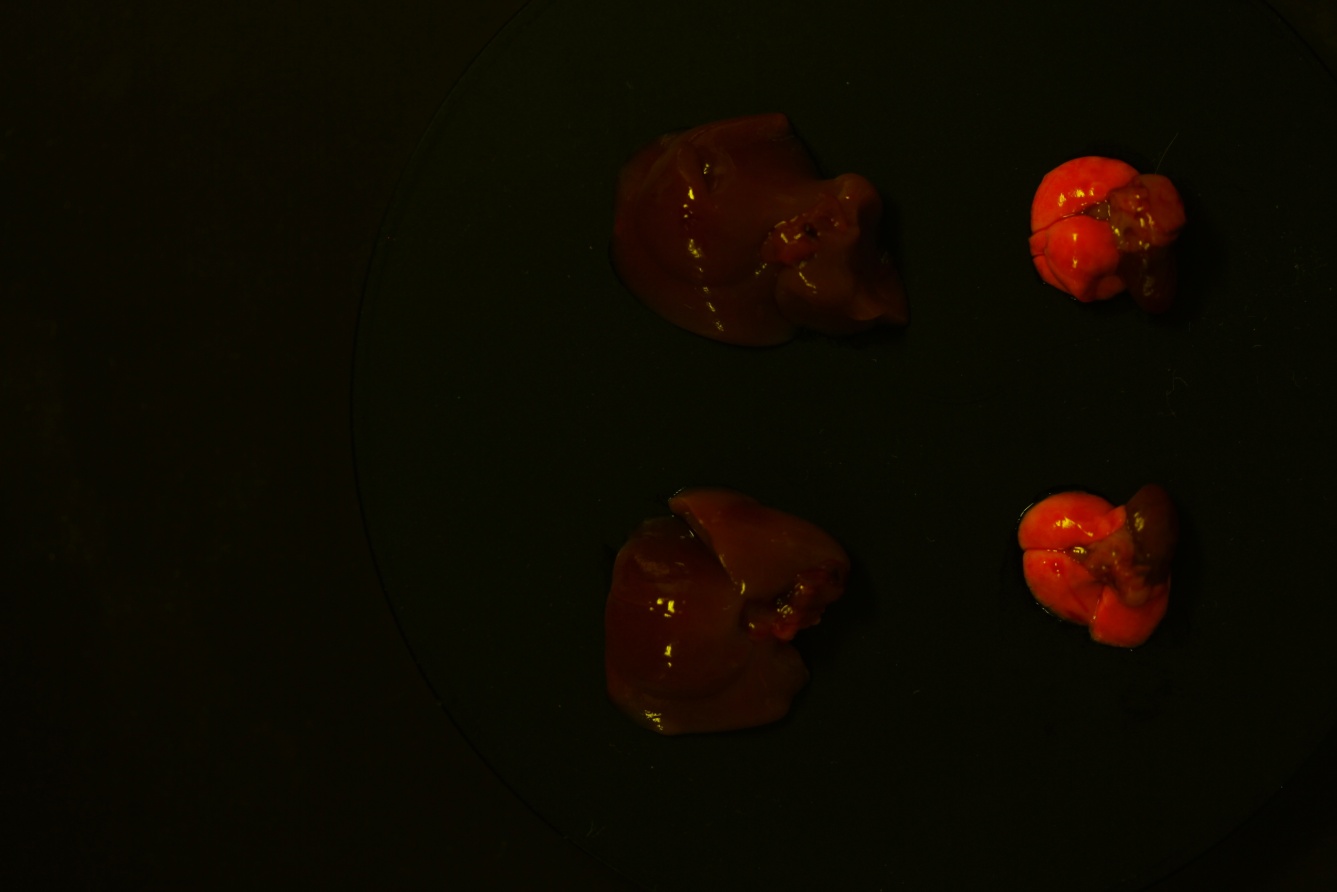


**GFP Imaging**

**GFP Imaging**

**Figure S4**

**A**

.

B
