# Supplementary material for: The Role of Interferon-γ Inducible Protein-10 in a Mouse Model of Acute Liver Injury Post Induced Pluripotent Stem Cells Transplantation
Source: PLoS One. 2012 Dec 5;7(12):e50577. doi: 10.1371/journal.pone.0050577 (PMC3515611; doi:10.1371/journal.pone.0050577)
Supplement: Table S1 — Primer sequences used in real time-PCR. (DOC) [file pone.0050577.s006.doc]

**Table S1.** Primer sequences used in real time-PCR

| Name | Sequences | | Length ID | |
| --- | --- | --- | --- | --- |
| IP-10 | | CGTCATTTTCTGCCTCATCCT  TGGTCTTAGATTCCGGATTCAG | | 227 NM_021274 |
| MIG | | ACTCAGCTCTGCCATGAAGTCCGC  AAAGGCTGCTCTGCCAGGGAAGGC | | 479 NM_008599 |
| ITAC | | ATGAACGGCTGCGACAAAGT  GCATGTTCCAAGACAGCAGA | | 225 NM_019494 |
| CXCR3 | | AGAATCATCCTGGTCTGAGACA  AAAGATAGGGCATGGCAGCTA | | 256 NM_009910 |
| IFN-α | | CCTGTGTGATGCAACAGGTC  TCACTCCTCCTTGCTCAATC | | 209 NM_010505 |
| IFN-γ | | TGATGGCCTGATTGTCTTTCAA  GGATATCTGGAGGAACTGGCAA | | 110 NM_0083337 |
| IFN-λ | | AGCTGCAGGCCTTCAAAAAG  TGGGAGTGAATGTGGCTCAG | | 244 NM_0010243673 |
| GAPDH | | TGTTGAAGTCACAGGAGACAACCT  AACCTGCCAAGTATGATGACATCA | | 111 XR_030913 |
