# Supplementary material for: The Role of Interferon-γ Inducible Protein-10 in a Mouse Model of Acute Liver Injury Post Induced Pluripotent Stem Cells Transplantation
Source: PLoS One. 2012 Dec 5;7(12):e50577. doi: 10.1371/journal.pone.0050577 (PMC3515611; doi:10.1371/journal.pone.0050577)
Supplement: Table S2 — Organ distribution of iPS injected into CCl4-injured mice. (DOC) [file pone.0050577.s007.doc]

**Table S2.** Organ distribution of iPS injected into CCl_4_-injured mice

| Location | iPS (-)  % (n=5) | iPS (+)  % (n=5) | *p value* |
| --- | --- | --- | --- |
| Liver | 0.00±0.00 | 2.66±0.46 | 0.012 |
| Spleen | 0.14±0.03 | 4.74±1.68 | 0.028 |
| Lung | 0.03±0.02 | 2.21±0.25 | 0.00002 |
| Bone Marrow | 0.11±0.06 | 1.82±0.56 | 0.0159 |

Induced pluripotent stem (iPS) cells were labeled with fluorescence dye DiI with >99% labeling efficiency. The background fluorescence of the injured liver that received no iPS cells was used as reference. The percentages of positive cells derived from five independent experiments were used for analysis.
